# Skeletal age assessed by Greulich-Pyle: Intra-observer and inter-observer agreement among male pubertal tennis players

**DOI:** 10.1371/journal.pone.0307305

**Published:** 2025-01-22

**Authors:** Jorge M. Celis-Moreno, Diogo V. Martinho, Manuel J. Coelho-e-Silva, Isabel Fragoso, Luís P. Ribeiro, Élvio R. Gouveia, Tomas Oliveira, João Gonçalves-Santos, Oscar M. Tavares, Ricardo R. Cayolla, Pedro Duarte-Mendes, Jan M. Konarski, Robert M. Malina, Gillian K. Myburgh, Sean P. Cumming, Lauren B. Sherar

**Affiliations:** 1 University of Coimbra, FCDEF, Coimbra, Portugal; 2 Universidad Santo Tomas, Bogota, Colombia; 3 CIDAF (uid/ 04213/2020), University of Coimbra, Coimbra, Portugal; 4 Interactive Technologies Institute, LARSYS, Funchal, Portugal; 5 University of Lisbon, Faculty of Human Kinetics, Lisbon, Portugal; 6 CIPER (uid/0447/2020), University of Lisbon, Lisbon, Portugal; 7 School of Health, University of Algarve, Faro, Portugal; 8 Department of Physical Education and Sport, University of Madeira, Funchal, Portugal; 9 Coimbra Health School, Polytechnic Institute of Coimbra, Coimbra, Portugal; 10 REMIT (Research Centre on Economics Management and Information Technologies), University Portucalense, Porto, Portugal; 11 School of Education, Polytechnic Institute of Castelo Branco, Castelo Branco, Portugal; 12 Sport, Health & Exercise Research Unit (SHERU), Polytechnic Institute of Castelo Branco, Castelo Branco, Portugal; 13 Theory of Sports Department, Poznań University of Physical Education, Poznań, Poland; 14 Department of Kinesiology and Health Education, University of Texas at Austin, Austin, Texas, United States of America; 15 School of Public Health and Information Sciences, University of Louisville, Louisville, Kentucky, United States of America; 16 Fulham FC, London, United Kingdom; 17 Department of Health, University of Bath, Bath, United Kingdom; 18 School of Sport, Exercise & Health Sciences, Loughborough University, Loughborough, United Kingdom; University of Macerata: Universita degli Studi di Macerata, ITALY

## Abstract

The assessment of biological maturation is a central topic in pediatric exercise sciences. Skeletal age (SA) reflects changes in each bone of the hand and wrist from initial ossification to the adult state. This study examined intra-observer and inter-examiner agreement is Greulich-Pyle (GP) assessments of SA in 97 male tennis players 8.6–16.8 years of age. Two observers independently examined all films on two occasions using the GP method. The SA of each bone was evaluated. The mean and median of SAs assigned for each bone was the individual SA for each participant. The calculation was exclusively based on the bones that were not skeletally mature. Intra-observer mean differences were significant for several bones with better results by the experienced examiner (observer B). Comparisons between SA values of the two independent observers indicated significant differences for the ulna, metacarpals II and III, and distal phalanx V. Nevertheless, the magnitude of the bone-specific differences was small, perhaps trivial. Differences in individual SA values of the tennis players based on the non-mature bones of the hand-wrist were negligible based on the mean (0.04±0.39, t = 0.321, p = 0.749) or the median (0.05±0.58, t = 0.007, p = 0.994). Nevertheless, the current study confirmed examiners as a source of error in the estimation of SA using the Greulich-Pyle method and highlighted the importance of calculating SAs based on non-mature bones among adolescent players.

## Introduction

Growth refers to changes in body size with a focus on composition, proportions and shape. Biological maturation refers to the progress towards adult state and is not directly measured [1]. Growth and maturation different, though related, processes. Inter-individual differences in size and maturity status among youth of the same chronological age (CA) are considerable [2]. Additionally, maturity-associated variation explained functional capacities among adolescent tennis players [3]. In the context of youth sports, athletes are ordinarily grouped by CA for training and competition, although increasingly recommendations to group participants on the basis of estimated biological maturity status (for example, bio-banding) in an effort to accommodate maturity-associated variation in size and performance [2, 3]. Among judo athletes aged 9–16 years, for example, the contribution of CA, body size (stature, body mass), estimated fat mass and biological maturity status based on a predicted somatic indicator explained a substantial portion of inter-individual variance on several strength tests [4].

Maturity status refers to the state of maturation at the time of observation and is commonly assessed with several indicators [1, 5, 6]: sexual, somatic, skeletal and dental. Maturity timing, in contrast, refers to the age at which specific biological indicators occur, e.g., age at menarche or age at peak height velocity [1]. The tempo of maturation refers to the rate at which children and youth progress towards the mature state in the respective indicators [5]. The Study Group on Forensic Age Diagnostics [7] recommends the combination of a physical examination with anamnesis, an X-ray examination of the hand and a dental examination with evaluation of an orthopantomogram for age assessments of adolescents. Studies in the context of youth sports commonly classify youth as late (delayed), average (on time) or early (advanced) maturing, e.g., in soccer [8, 9], tennis [3, 10] and table tennis [11].

Secondary sex characteristics, specifically stages of pubic hair development, are often used as indicators of maturity status in studies of youth athletes [12–14]. The stages, however, are non-continuous and of course are limited to the pubertal years [1, 15]. On the other hand, skeletal (SA) is generally viewed as the best indicator of maturity status as it is applicable from childhood through adolescence [8, 10]. Concern for the alleged use of over-age players in some sports has led to the use of SA for the verification of chronological age [16]. Three methods for the assessment of SA are commonly used: Greulich-Pyle (GP), Tanner-Whitehouse (TW) and Fels; these protocols are based on hand-wrist radiographs. Details of the specific methods are summarized elsewhere [1, 16]. Nevertheless, as a radiograph requires a small amount of radiation, SA assessment is often considered invasive [17, 18]. Recent studies used magnetic resonance imaging to estimate bone age from the carpal bones [19] and, alternatively, the metaphyseal–epiphyseal fusion of long bones [19]. Although interesting, magnetic resonance imaging is rarely available.

The GP method, also called “the Atlas” method, was developed on a sample of American boys and girls in the state of Ohio who were followed from birth to mature state. The method requires the evaluation of each of the 30 bones relative to sex-specific standard plates from infancy through adolescence [20]. Unfortunately, the youth soccer literature is generally lacking in the details of how the GP method is applied in assessment of SA based on the 30 bone-specific SAs [21]. On the other hand, a recent study [22] recommended that the SAs of only non-mature bones with the GP protocol be used in calculating the SA for individuals. In the context of the preceding, this study considers intra-observer and inter-examiner agreement in the assessment of SAs with the GP method. It was hypothesized that agreement rates will vary depending on specific bones. Additionally, it was hypothesized that the overall SA of a tennis player exclusively based on the non-mature bones of the hand-wrist fluctuates depending of the calculations using the mean or the median SA values of non-mature bones.

## Material and methods

### Procedures

The project was approved by the *Ethics Committee in Sports Sciences* at the *University of Coimbra* (CE/FCDEF-UC/00122014). Informed consent was signed by parents or legal guardians of the participants before starting data collection. Players were verbally informed about the procedures, objectives, benefits and risks of the study, and also informed that they could withdraw from the study at any time. This procedure was done in accordance with the Declaration of Helsinki for human studies of the World Medical Association [23].

### Sample

The sample included 97 male tennis players aged 8.69–16.84 years who were registered in a competitive tennis club for at least one complete season and were <17 years of age. At the time of data collection, all players trained 3–4 sessions per week. CA was calculated as birth date minus the date of the visit to the clinic for the radiograph. The measurements were taken between May 2014 and September 2019.

### Skeletal age

A radiograph of the left hand-wrist was taken by a certified technician on the same day as anthropometric dimensions were measured. Each film was examined using the GP protocol [20]. The method requires the evaluation of radius, ulna, eight carpals (capitate, hamate, triquetral, pisiform, lunate, scaphoid, trapezium, trapezoid), five metacarpals, adductor sesamoid, five proximal phalanges, four middle phalanges (II, III, IV, V), and five distal phalanges. It involves matching each bone of the hand-wrist on the radiograph of the individual with the sex-specific plates of the GP Atlas; the SA of closest plate of the Atlas to which each bone of the subject matches is the assigned SA for the bone. For example, if the image of the radius of a 13-year-old male tennis player matched the standard plate of the Atlas for an 11-year-old boy, the SA of the radius of the participant is 11 years. The process is repeated for all bones of the hand-wrist. The SA for the player was estimated from the SAs assigned to all non-mature bones according to the following options. Firstly, overall SA was estimated as the mean of the bone-specific SAs for the non-mature bones. Subsequently, calculations were repeated using the median.

### Analyses

All films were independently examined on two occasions by two observers: A and B. Observer A had a 45-hour education and preparation which included the assessment of 100 radiographs using the GP method. Observer B was an experienced examiner who already completed more than 1000 SA assessments with the GP method. (The latter also had considerable experience with SA assessments using Tanner-Whitehouse and Fels methods).

As noted, each film was evaluated twice by the two observers. Intra-observer error was obtained on the difference between SAs assigned to individual bones in two assessments (moment 2 minus moment 1). Paired student t-tests were used to evaluate mean differences between time-moments separately for observer A and observer B. Inter-observer error was calculated as the differences between examiners at time moment 2 (observer A minus observer B) using paired student t-tests. The analyses were complemented with calculations of magnitude effect using d-values [24] which were interpreted as follows [25]: d<0.2 (trivial), 0.2≤d<0.6 (small), 0.6≤d<1.2 (moderate), 1.2≤d<2.0 (large), 2.0≤d<4.0 (very large) and d≥4.0 (nearly perfect). Intra-class correlation coefficients between observers at time-moment 2 was determined for the assigned SA of the tennis players. The preceding was repeated with the SA calculated as the mean of the bone-specific SAs for the non-mature bones and as the median. For the analyses, SPSS (version 27: IBM Company, Armonk, NY, USA) was used. Inter-observer individual differences were plotted using Bland-Altman analyses (GraphPad Prism V.7 for Windows, GraphPad Software, San Diego, California USA, www.graphpad.com) separately for each option used to calculate assigned SA from bone-specific SAs.

## Results

Descriptive statistics for CA, stature and body mass of the tennis players are summarized in Table 1, while the frequencies of bones that are classified as, respectively, mature or not-mature and the descriptive statistics for each of the 30 bones are summarized (range, median, mean and standard deviation) for time-moment 1 and time-moment 2 are summarized in Table 2 for observer A and in Table 3 for observer B. For each observer, the highest frequencies of bones classified as already mature occur for the carpals and distal phalanges. No player was rated as mature for the radius. For observer A, mean differences between time-moments are significant for 17 of the 30 bones: radius, capitate, hamate, triquetral, trapezium, metacarpal II-V, proximal phalange II-V, medial phalange II-V. For observer B, in contrast, only four bones present significant mean differences by time-moments: lunate, trapezium, medial phalanges II and III. Inter-observer differences in bone SAs of the tennis players at time moment 2 are summarized in Table 4. Albeit trivial, significant mean differences between examiners are apparent for the ulna, metacarpals II and III, and distal phalanx V.

**Table 1:** Descriptive statistics for chronological age, stature and body mass for the total sample of 97 tennis players.

Variable, units	Minimum	Maximum	Mean		Standard deviation
			Mean	Standard error of the mean	
Chronological age, years	8.69	16.84	12.8	0.2	1.8
Stature, cm	124.4	193.1	158.6	1.4	13.7
Body mass, kg	25.5	85.5	48.6	1.4	13.5

**Table 2:** Intra-observer errors for the SAs of individual bones of the hand-wrist of the tennis players assigned by observer A.

	time-moment 1					time-moment 2					paired t-test		
	mature (n)	pre-mature				mature (n)	pre-mature						
		n	range	median	mean ± sd		n	range	median	mean ± sd	df	t	p
Radius	0	97	8.17–18.0	12.5	13.07 ± 2.28	0	97	8.17–18.0	13.0	13.14 ± 2.25	96	1.983	0.050
Ulna	2	95	8.50–17.0	13.0	12.86 ± 2.07	2	95	8.50–17.0	13.0	12.88 ± 2.06	94	0.023	0.982
Capitate	40	57	7.00–14.0	12.5	11.86 ± 1.55	38	59	7.00–14.0	12.5	11.79 ± 1.47	56	2.179	0.034
Hamate	40	57	9.00–14.0	12.5	11.87 ± 1.45	38	59	9.00–14.0	12.5	11.80 ± 1.37	56	2.320	0.024
Triquetral	40	57	8.00–14.0	12.5	11.89 ± 1.44	38	59	8.00–14.0	12.5	11.80 ± 1.42	56	2.015	0.049
Pisiform	40	57	8.00–14.0	12.5	12.07 ± 1.40	38	59	9.00–14.0	13.0	12.28 ± 1.23	56	1.669	0.101
Lunate	40	57	8.67–14.0	12.5	11.85 ± 1.43	38	59	8.67–14.0	12.5	11.82 ± 1.34	56	1.611	0.113
Scaphoid	40	57	8.17–14.0	12.5	11.80 ± 1.51	38	59	8.17–14.0	12.5	11.84 ± 1.39	56	0,331	0,742
Trapezium	40	57	8.17–14.0	12.5	11.89 ± 1.54	38	59	8.17–14.0	12.5	11.79 ± 1.44	56	2.421	0.019
Trapezoid	40	57	8.17–14.0	12.5	11.86 ± 1.50	38	59	8.17–14.0	12.5	11.85 ± 1.40	56	1.090	0.280
AS	40	57	8.17–12.5	11.5	11.31 ± 1.18	40	57	8.17–12.5	11.5	11.31 ± 1.18	‡	‡	‡
MET I	12	85	8.00–15.0	12.5	12.33 ± 1.86	10	87	9.00–15.0	12.5	12.46 ± 1.81	84	1.045	0.299
MET II	4	93	8.00–16.0	13.0	12.55 ± 2.12	8	89	9.00–16.0	12.5	12.57 ± 1.97	88	3.083	0.003
MET III	4	93	8.00–16.0	13.0	12.54 ± 2.11	9	88	9.00–16.0	12.5	12.52 ± 1.95	87	3.114	0.003
MET IV	4	93	8.00–16.0	13.0	12.52 ± 2.12	9	88	8.00–16.0	12.5	12.50 ± 1.94	87	3.284	0.001
MET V	4	93	8.00–16.0	13.0	12.54 ± 2.09	9	88	8.00–16.0	12.5	12.49 ± 1.94	87	2.781	0.007
PP I	6	91	8.00–16.0	12.5	12.73 ± 2.11	6	91	7.00–16.0	12.5	12.82 ± 2.06	88	1.779	0.079
PP II	3	94	8.00–16.0	12.5	12.78 ± 2.18	5	92	7.00–16.0	12.5	12.87 ± 2.04	91	2.422	0.017
PP III	3	94	8.00–16.0	12.5	12.78 ± 2.18	5	92	7.00–16.0	12.5	12.86 ± 2.02	91	2.034	0.045
PP IV	3	94	8.00–16.0	12.5	12.78 ± 2.18	5	92	8.00–16.0	12.5	12.85 ± 2.02	91	2.268	0.026
PP V	3	94	8.00–16.0	12.5	12.78 ± 2.18	5	92	8.00–16.0	12.5	12.85 ± 2.06	91	2.669	0.009
MP II	6	91	7.83–16.0	12.2	12.43 ± 2.30	10	87	8.75–16.0	12.2	12.41 ± 2.12	86	2.457	0.016
MP III	10	87	7.83–16.0	12.2	12.27 ± 2.21	12	85	8.75–16.0	12.2	12.34 ± 2.07	84	2.578	0.012
MP IV	11	86	7.83–16.0	12.2	12.19 ± 2.21	11	86	8.75–16.0	12.2	12.33 ± 2.13	84	2.400	0.019
MP V	8	89	7.83–16.0	12.2	12.28 ± 2.35	10	87	7.50–16.0	12.2	12.35 ± 2.20	86	2.385	0.019
DP I	4	93	8.00–15.0	12.5	12.50 ± 1.92	8	89	9.00–15.0	12.5	12.45 ± 1.97	88	1.014	0.313
DP II	21	76	8.00–15.0	12.5	12.02 ± 1.71	21	76	9.00–15.0	12.5	12.05 ± 1.84	74	0.376	0.708
DP III	12	85	8.00–15.0	12.5	12.37 ± 1.81	16	81	9.00–15.0	12.5	12.21 ± 1.88	78	0.486	0.629
DP IV	20	77	8.00–15.0	12.5	12.09 ± 1.71	20	77	9.00–15.0	12.5	12.09 ± 1.87	75	0.093	0.926
DP V	20	77	8.00–15.0	12.5	12.03 ± 1.75	20	77	9.00–15.0	12.5	12.07 ± 1.87	75	0.545	0.587

AS (Adductor Sesamoid); MET (metacarpal); PP (proximal phalange); MP (medial phalange); DP (distal phalange)

‡ (paired t-test cannot be computed because the standard error of the difference is zero); sd (standard deviation); df (degrees of freedom).

**Table 3:** Intra-observer errors for the SAs of individual bones of the hand-wrist of the tennis players assigned by observer B.

	time-moment 1					time-moment 2					paired t-test		
	mature (n)	pre-mature				mature (n)	pre-mature						
		n	range	median	mean ± sd		n	range	median	mean ± sd	df	t	p
Radius	0	97	8.17–18.0	13.0	13.15 ± 2.25	0	97	8.17–18.0	13.0	13.18 ± 2.28	96	0.778	0.438
Ulna	2	95	7.00–17.0	12.5	12.72 ± 1.99	2	95	7.50–17.0	12.5	12.72 ± 1.99	94	0.244	0.808
Capitate	39	58	7.00–14.0	11.5	11.68 ± 1.41	38	59	7.00–14.0	11.5	11.68 ± 1.48	57	0.927	0.358
Hamate	39	58	9.00–14.0	11.5	11.87 ± 1.45	39	58	8.00–14.0	11.5	11.74 ± 1.41	57	1.224	0.226
Triquetral	39	58	9.00–14.0	12.5	11.94 ± 1.19	39	58	9.00–14.0	12.5	11.94 ± 1.17	57	0.184	0.855
Pisiform	39	58	9.00–14.0	13.0	12.37 ± 1.22	39	58	9.00–14.0	13.0	12.37 ± 1.22	57	‡	‡
Lunate	39	58	8.67–14.0	12.5	11.87 ± 1.27	39	58	8.67–14.0	12.5	11.96 ± 1.25	57	2.351	0.022
Scaphoid	39	58	8.17–14.0	11.5	11.79 ± 1.39	37	60	8.17–14.0	12.5	11.93 ± 1.42	57	1.321	0.192
Trapezium	39	58	8.17–14.0	11.5	11.62 ± 1.52	39	58	8.17–14.0	11.5	11.71 ± 1.45	57	2.375	0.021
Trapezoid	39	58	8.17–14.0	12.5	11.83 ± 1.38	39	58	8.17–14.0	12.5	11.80 ± 1.36	57	0.830	0.410
AS	40	57	8.17–12.5	11.5	11.31 ± 1.18	40	57	8.17–12.5	11.5	11.31 ± 1.18	‡	‡	‡
MET I	13	84	9.00–15.0	12.5	12.35 ± 1.76	14	83	9.00–15.0	12.5	12.32 ± 1.74	83	0.445	0.657
MET II	9	88	9.00–16.0	12.5	12.73 ± 1.71	10	87	9.00–16.0	12.5	12.70 ± 1.66	83	0.300	0.765
MET III	10	87	9.00–16.0	12.5	12.66 ± 1.70	10	87	9.00–16.0	12.5	12.66 ± 1.70	87	0.000	1.000
MET IV	10	87	8.00–16.0	12.5	12.54 ± 1.78	10	87	8.00–16.0	12.5	12.54 ± 1.70	87	0.185	0.854
MET V	9	88	8.00–16.0	12.5	12.62 ± 1.77	10	87	8.00–16.0	12.5	12.60 ± 1.72	88	0.464	0.644
PP I	12	85	7.00–16.0	12.5	12.60 ± 1.84	9	88	7.00–16.0	12.5	12.55 ± 1.82	88	1.915	0.059
PP II	12	85	7.00–16.0	13.0	12.47 ± 1.98	12	85	7.00–16.0	13.0	12.47 ± 1.99	85	0.000	1.000
PP III	12	85	7.00–16.0	13.0	12.88 ± 1.78	12	85	7.00–16.0	13.0	12.68 ± 1.77	85	0.000	1.000
PP IV	12	85	8.00–16.0	13.0	12.70 ± 1.75	12	85	8.00–16.0	13.0	12.69 ± 1.74	85	0.332	0.741
PP V	12	85	8.00–16.0	13.0	12.72 ± 1.75	12	85	8.00–16.0	13.0	12.70 ± 1.73	85	0.904	0.369
MP II	12	85	8.75–16.0	12.2	12.37 ± 1.94	12	85	8.75–16.0	12.2	12.32 ± 1.91	85	2.233	0.028
MP III	12	85	8.75–16.0	12.2	12.36 ± 1.92	12	85	8.75–16.0	12.2	12.32 ± 1.90	85	1.725	0.088
MP IV	11	86	8.75–16.0	12.2	12.32 ± 2.01	11	86	8.75–16.0	12.2	12.31 ± 1.98	86	0.106	0.916
MP V	11	86	8.50–16.0	12.2	12.17 ± 2.07	11	86	8.50–16.0	12.2	12.18 ± 2.04	86	0.314	0.755
DP I	20	77	9.00–15.0	11.5	12.03 ± 1.57	8	89	9.00–15.0	11.5	12.02 ± 1.58	77	1.000	0.320
DP II	18	79	9.00–15.0	11.5	12.08 ± 1.62	20	77	9.00–15.0	11.5	12.06 ± 1.63	79	1.349	0.181
DP III	20	77	9.00–15.0	11.5	12.16 ± 1.49	20	77	9.00–15.0	11.5	12.15 ± 1.49	77	1.000	0.320
DP IV	18	79	9.00–15.0	11.5	12.11 ± 1.63	18	79	9.00–15.0	11.5	12.11 ± 1.63	79	‡	‡
DP V	16	81	9.00–15.0	11.5	12.03 ± 1.74	17	80	9.00–15.0	11.5	12.00 ± 1.73	80	0.276	0.783

AS (Adductor Sesamoid); MET (metacarpal); PP (proximal phalange); MP (medial phalange); DP (distal phalange); ‡ (paired t-test cannot be computed because the standard error of the difference is zero); sd (standard deviation); df (degrees of freedom).

**Table 4:** Inter-observer differences in assigned SAs of the tennis players at time moment 2.

	mean difference (observer B–observer A)		paired t-test		effect size	
	value	(95%CI)	t-value	p	d-value	(qualitative)
Radius	0.04	(-0.11 to 0.19)	0.530	0.598	0.018	(trivial)
Ulna	-0.16	(-0.29 to -0.02)	2.330	0.022	0.079	(trivial)
Capitate	-0.13	(-0.37 to 0.11)	1.087	0.282	0.007	(trivial)
Hamate	-0.04	(-0.27 to 0.18)	0.382	0.704	0.044	(trivial)
Triquetral	0.16	(-0.06 to 0.39)	1.425	0.160	0.108	(trivial)
Pisiform	0.10	(-0.07 to 0.28)	1.169	0.247	0.074	(trivial)
Lunate	0.16	(-0.01 to 0.32)	1.832	0.072	0.887	(large)
Scaphoid	0.04	(-0.13 to 0.22)	0.489	0.626	0.065	(trivial)
Trapezium	-0.06	(-0.27 to 0.15)	0.582	0.563	0.056	(trivial)
Trapezoid	-0.03	(-0.23 to 0.17)	0.258	0.797	0.037	(trivial)
AS	‡	‡	‡	‡	‡	‡
MET I	-0.02	(-0.15 to 0.11)	0.278	0.782	0.079	(trivial)
MET II	0.21	(0.05 to 0.36)	2.660	0.009	0.072	(trivial)
MET III	0.18	(0.03 to 0.34)	2.331	0.022	0.077	(trivial)
MET IV	0.08	(-0.07 to 0.23)	1.044	0.299	0.022	(trivial)
MET V	0.11	(-0.03 to 0.26)	1.537	0.128	0.060	(trivial)
PP I	-0.04	(-0.21 to 0.13)	0.516	0.607	0.140	(trivial)
PP II	-0.14	(0.31 to 0.04)	1.560	0.122	0.200	(small)
PP III	-0.08	(-0.08 to 0.25)	0.985	0.328	0.095	(trivial)
PP IV	0.10	(-0.06 to 0.26)	1.215	0.228	0.085	(trivial)
PP V	0.11	(-0.60 to 0.27)	1.271	0.207	0.079	(trivial)
MP II	-0.01	(-0.15 to 0.13)	0.143	0.887	0.045	(trivial)
MP III	-0.02	(-0.16 to 0.11)	0.319	0.750	0.010	(trivial)
MP IV	-0.02	(-0.15 to 0.12)	0.263	0.793	0.010	(trivial)
MP V	-0.13	(-0.29 to 0.02)	1.673	0.098	0.081	(trivial)
DP I	-0.03	(-0.20 to 0.14)	0.366	0.716	0.242	(small)
DP II	-0.11	(-0.28 to 0.06)	1.210	0.230	0.006	(trivial)
DP III	0.09	(-0.10 to 0.28)	0.975	0.333	0.035	(trivial)
DP IV	-0.05	(-0.23 to 0.10)	0.607	0.545	0.011	(trivial)
DP V	-0.20	(-0.38 to -0.01)	2.117	0.038	0.039	(trivial)

AS (Adductor Sesamoid); MET (metacarpal); PP (proximal phalange); MP (medial phalange); DP (distal phalange); ‡ Paired t-test cannot be computed because the standard error of the difference is zero; 95%CI (95% confidence interval).

**Fig 1:**
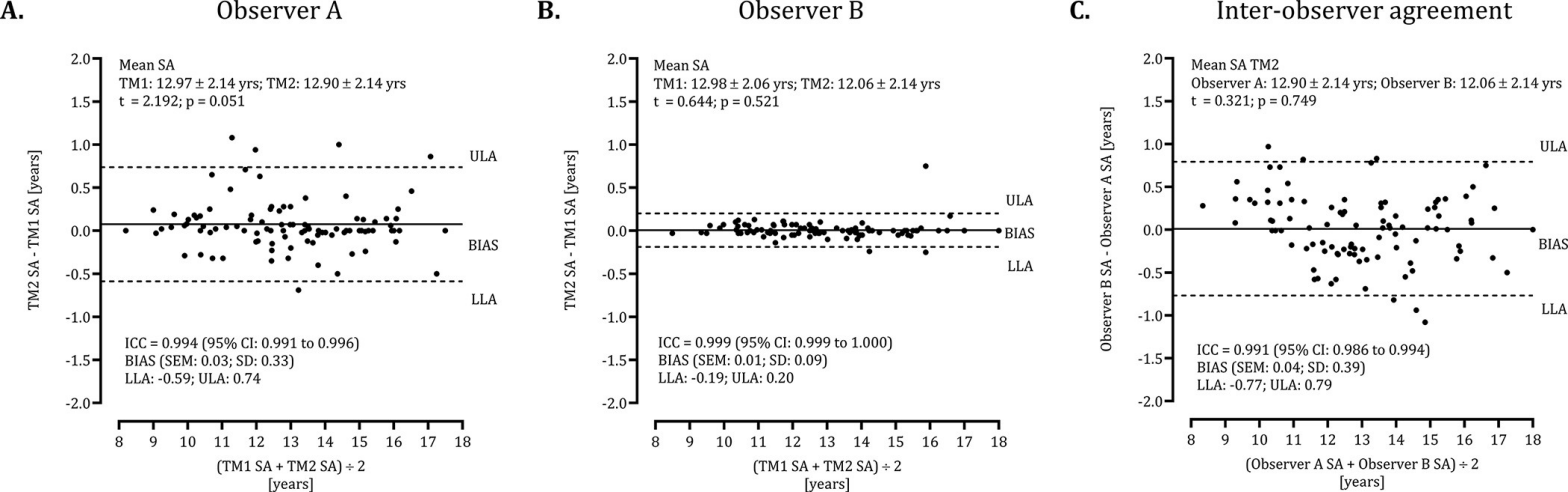
Bland-Altman analysis of intra-individual differences and inter-observer agreement for the assigned SA based on means of the SAs assigned to each bone with the Greulich-Pyle method.

**Fig 2:**
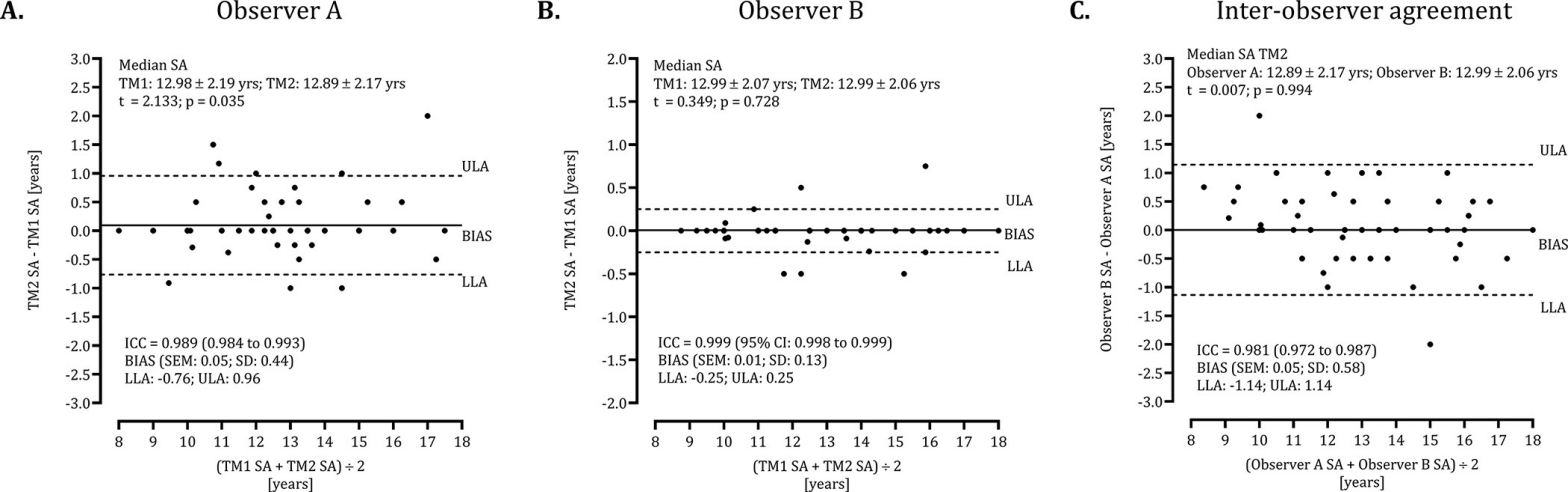
Bland-Altman analysis of intra-individual differences and inter-observer agreement for the assigned SA based on medians of the SAs assigned to each bone with the Greulich-Pyle method.

SAs for each player were calculated from the respective bone-specific SAs. Intra-individual differences and inter-observer agreement for the assigned SA based on means and on the medians of the SAs assigned to each bone with the GP method are illustrated in Figs 1 and 2, respectively. For estimated SAs based on means of the assigned SAs for each bone (Fig 1), ICC values are high for both observers A: 0.994 and B: 0.999 (Fig 1). For estimated SAs based on medians of the assigned SAs for each bone (Fig 2), in contrast, ICC values are slightly lower for observer A (0.989) compared to observer B (0.999). Nevertheless, the difference between observers is negligible when using either the means (BIAS: 0.04± 0.39, t = 0.321, p = 0.749) or the medians (BIAS: 0.05± 0.58, t = 0.007, p = 0.994).

## Discussion

The maturation of the hand-wrist bones follows universal sequences of changes in the bone tissue that permits the assessment of bone-specific SAs [1, 20]. Briefly, each bone is characterized by a sequence from the immature to the mature state. The aim of the present study was to determine intra-observer and inter- observer agreement of estimates of SA based on GP method among male tennis players 8 through 16 years of age. Although the assessments of observer A were characterized by significant mean differences for repeated measurements on 17 of the 30 bones, the magnitude of the errors associated to observer A did not show a specific trend, i.e., replicate estimates were not systematically higher or lower than initial assessments. As expected, repeated measurements were slightly more stable for observer B who had considerably more experience in the assessment of SA. Although bone-specific intra-observer errors, the overall SA assigned for the tennis players did not differ between examiners.

Long bones are characterized by the epiphyseal-diaphyseal junction which is a major source of information for the assessment of SA, especially during late adolescent years when fusion of the epiphysis and diaphysis of long bones ordinarily begins. On the other hand, the carpal bones attain the mature state at earlier ages compared to radius, ulna, metacarpals and phalanges. The shape of the bones (for example the convexity or concavity of the margins) is also an indicator for the estimating stage of maturity of the respective bones [1, 26]. The tennis players in the present classified as average in skeletal maturity status tended to have SAs that corresponded to the reference sample used to develop the GP method. Similarly, the tennis players classified as early and late maturing had SAs that varied in timing relative to the criteria of the GP method. Note, however, that a male tennis player with an SA of 14.5 years based on the GP method does not necessarily correspond to a player characterized with an SA of 14.5 years based on the Tanner-Whitehouse and Fels methods of SA assessment [18].

The GP method, often called the Atlas method, uses sex-specific criteria that vary with CA to evaluate the status of the hand-wrist 30 bones. In fact, the GP was developed based on changes in the process of maturation among 29 boys and 31 girls followed longitudinally from childhood through adolescence [20]. The protocol is based on a series of illustrations of radiographs that allow the identification of the approximate standards for the specific bone under examination. The examiner makes assessments based on pictorial standards. However, in the calculation of the final SA, the GP method is lacking in the details of how the individual bones classified as mature at different CAs should be considered. It is also not clear whether to use the mean or the median to derive the SA based on the maturity status (i.e., SA) of the specific bones. The preceding limitations were addressed in the Tanner-Whitehouse (TW) method. Briefly, the 20 bones assessed with the TW method are assigned a specific stage to which a sex-specific score is assigned; the summed score for the 20 bones is the converted to an SA using scales for boys and girls. The initial version of the TW method [27] was based on a large sample of British children from public schools who were born between 1940 and 1955. The second version of the TW method [28] created three concurrent systems to determine SA: 20-bones, RUS (radius, ulna and long bones) and Carpal (based on 7 carpals, excluding the pisiform). The most recent version of the TW methods is limited to RUS and Carpal SAs [1, 16, 18]. In contrast to TW method, the FELS method [29] includes the radius, ulna, seven carpals, and epiphyses of metacarpals and phalanges of the first (thumb), third and fifth digits of the hand-wrist skeleton to derive an SA using sex-specific criteria for girls and boys, in addition to the presence or absence of the pisiform and adductor sesamoid, and measurements of epiphyseal and metaphyseal widths. The Fels protocol provides SA and associated error based on specific software (Felsw 1.0).

An earlier study of the reliability of SA assessments using the GP method considered the influence of variation in the prior experiences of those assessing the radiographs, specifically comparisons of experienced examiners with less experienced peers. The study noted that mean SAs based on multiple assessments by paired observers were more stable in reproducibility than paired assessments of the same observer [30]. When the less experienced observer had the opportunity to contrast discrepancies with an expert, replicability was improved [31]. It was subsequently demonstrated [32] that when bone-specific SAs were obtained by an observer to whom the whole hand- wrist was visible, the SAs were influenced by the scores already made for bones in the same row. It was thus recommended that assessments should be done with only one bone visible at a time. Accordingly, examiners would be instructed to assess the bones of the hand-wrist in such a way that, for example, the rating of metacarpal V would not be influenced by the ratings of metacarpals II-IV.

A recent study of 100 Portuguese female soccer players 12.0–16.7 years of age concluded that the GP method was satisfactorily reproducible [22]. Intra-observer agreements fluctuated 90% for trapezium and 95% for trapezoid regarding the novice examiner. The experienced observer consistently attained higher rates for all bones. Nevertheless, the preceding rates, inter-observer agreements emerged as problematic for the scaphoid (81%) and trapezium (84%). Similarly, others have noted that the carpals were seemingly more difficult to assess and generated larger inter-individual variability compared to the long bones [33]. An additional methodological consideration that is not clear with the GP method is the derivation of the final SA.

Among pubertal female soccer players aged 12.0–16.7 years [22], the mean of the 30 bones (non-mature and skeletally mature) was compared to the mean of only the non-mature bones corresponded to trivial differences (less experience observer: 14.20±0.36 years for 30 bones, 14.24±0.077 years for the non-mature bones; expert: 14.18±0.32 and 14.17±0.74, respectively). Based on the medians in the cited study [22], the SA of the sample slightly increased to 14.56±0.61 years for the two examiners; differences between observers were negligible. With increasing CA, it is expected that calculations of SA would be based on a smaller number of bones. Indeed, when using Fels protocol, the automated error tends to increase with age from 0.29 years to 0.54 years, respectively, from 1 to 18 years of age [29].

During effective playing time in the best-of-three-sets, players often sprint 3 meters per shot, 8 to 12 meters during a point, and 300 to 500 high-intensity attempts. The development of sprinting performance in elite young Dutch tennis players was explained by longitudinal changes in body size and lower limb strength with negligible differences in 5-m sprints by players contrasting in maturity status given by a somatic indicator [34]. By inference, coaches need to be aware of variation among players and methods of maturity assessment in an effort to adequately assess maturity status and to interpret the performance of players relative to training, body size and biological maturation. Based on GP SA assessments, for example, elite French youth soccer players assessed as early maturing were18 cm taller and 20 kg heavier compared to late maturing players and also attained better scores on isokinetic strength and sprint tests [35].

The current study is limited to males and the results should not be generalized to younger ages. There is also a need for further studies of variation in SA assessments with different methods within specific CA groups of athletes spanning a broader range. This would permit more detailed assessment of variation in SA assessments with the GP method, specifically during the early stages of bone development. This would also require substantially larger samples and several observers. Caution is warranted when comparing the results of studies of youth athletes that assess maturity status with different methods and efforts to relate the observations to athletes in different sports and also to the general population.

As the issue of low level radiation exposure with radiographs is a concern, studies of youth athletes using other methods of SA assessment merit attention, although data are not extensive. For example, ultrasound estimates of SA based on the distal epiphyses of the radius and ulna (scaled to the GP method) were similar to CA among 11-year-old soccer players, but in advance of CA among 16 year old players [36]. Estimated SA based on the automated BoneXpert method (scaled to the GP method) was, on average, similar to mean CA among soccer players 14 years of age [37]. Corresponding data are apparently lacking for youth tennis players.

## Conclusion

Results of this study of youth tennis players suggest that the assessment of maturity status of the carpal bones is more problematic than assessments of the long bones using the GP method. Based on the current observations, it is also suggested that application of the GP method should be exclusively limited to non-mature bones, specifically when assessing older adolescents who have likely attained mature status of the carpals or round bones. Moreover, the calculation of overall SA based on the means or medians of the bone-specific SAs did not differ.

## Supporting information

S1 Data (XLSX)
